# Bone Regeneration Effect of Hyperbaric Oxygen Therapy Duration on Calvarial Defects in Irradiated Rats

**DOI:** 10.1155/2019/9051713

**Published:** 2019-04-02

**Authors:** Kyeong-Mee Park, Changdae Kim, Wonse Park, Young-Bum Park, Moon-Kyu Chung, Sungtae Kim

**Affiliations:** ^1^Department of Advanced General Dentistry, Yonsei University College of Dentistry, Seoul, Republic of Korea; ^2^Department of Prosthodontics, Yonsei University College of Dentistry, Seoul, Republic of Korea; ^3^Department of Periodontology, Dental Research Institute, Seoul Nation University School of Dentistry, Seoul, Republic of Korea

## Abstract

**Objective:**

In this study, we evaluated changes in bone remodeling in an irradiated rat calvarial defect model according to duration of hyperbaric oxygen therapy.

**Materials and Methods:**

The 28 rats were divided into four groups. Radiation of 12 Gy was applied to the skull, and 5-mm critical size defects were formed on both sides of the skull. Bone grafts were applied to one side of formed defects. From the day after surgery, HBO was applied for 0, 1, and 3 weeks. At 6 weeks after bone graft, experimental sites were removed and analyzed for radiography, histology, and histomorphometry.

**Results:**

Micro-CT analysis showed a significant increase in new bone volume in the HBO-3 group, with or without bone graft. When bone grafting was performed, BV, BS, and BS/TV all significantly increased. Histomorphometric analysis showed significant increases in %NBA and %BVN in the HBO-1 and HBO-3 groups, regardless of bone graft.

**Conclusion:**

Hyperbaric oxygen therapy was effective for bone regeneration with only 1 week of treatment.

## 1. Introduction

For treatment of head and neck tumors, radiation therapy is used in conjunction with surgical resection for complete tumor tissue killing. Irradiation uses ionizing radiation to induce cell death by damaging the genes of fissionable cells, which affects not only the tumor but also surrounding normal tissue. In response to this tissue reaction, oral mucositis, changes in taste, decreased salivation, radiation caries, and radiation osteonecrosis may occur in the oral cavity [1].

Irradiation reduces the ability to regenerate bone within the irradiation range [2]. Bone exposed to radiation is in a hypocellular, hypovascular, hypoxic environment that is unfavorable compared to the preirradiation condition for bone regeneration or remodeling. Cumulative damage from radiation is in the form of osteoradionecrosis (ORN) [3]. In general, the higher the radiation dose, the higher the probability that radiation osteonecrosis will occur [4]. Trauma to irradiated bone may also cause radiation necrosis [2]. The most frequent causes of trauma are extraction, ulcers caused by dentures, and periapical periodontitis [5]. In patients who receive more than 65 Gy of radiation, the likelihood of ORN during extraction after irradiation is two times higher than that in the preirradiation state [6].

Reconstruction of bone defects in patients who have undergone radiation is an indispensable procedure for the patient's functional and aesthetic restoration. Reconstruction of defects can restore function such as chewing, pronunciation, and swallowing; aesthetic improvement helps patients return to their daily lives. However, patients who have undergone irradiation do not have complete natural healing due to deficient bone-healing ability [1]. Therefore, in an impaired osteogenic condition, hyperbaric oxygen therapy may be combined with bone grafts to reconstruct defects [7, 8].

Hyperbaric oxygen therapy (HBO) is an adjunct therapy to overcome impaired osteogenic conditions that affect bone regeneration such as radiation [7, 8]. After a report that ORN was treated with HBO [9], HBO has been used by many clinicians as an adjunct treatment for ORN [10].

The anticipated effect of HBO is reducing bone resorption caused by hypovascularization by injecting oxygen at high pressure to resolve the hypoxic state of the bone due to poor vascularization [11]. Physiologically, the amount of hemoglobin in the blood is fixed, so the supply of oxygen is limited. However, since gas solubility is high in a high-pressure state, oxygen can be directly transferred into plasma. Theoretically, at 100% oxygen delivery at 2.5 ATA, oxygen in the plasma can vary by about 17 times compared to 21% oxygen at 1 ATA [12]. This level can meet the oxygen demand in the bone [12].

Hypoxia is a necessary process to initiate angiogenesis, but a prolonged hypoxic state adversely affects the healing process [13]. Persistent hypoxia inhibits fibroblast differentiation, collagen synthesis, and granulation tissue formation [3]. HBO contributes to the healing process by eliminating hypoxia. In addition, HBO promotes alkaline phosphatase activity during bone regeneration and HBO contributes to osteoblast activity and angiogenesis upon distraction of irradiated bone [14, 15]. The purpose of this study was to evaluate the effect of HBO on the duration of hyperbaric oxygen therapy in the presence or absence of bone graft.

## 2. Materials and Methods

### 2.1. Animals

This study used 28 healthy Sprague-Dawley rats (8-week-old males). Animal selection, preparation, and surgical protocols were conducted according to the Association for Assessment and Accreditation of Laboratory Animal Care guidelines and approved by the International Animal Care and Use Committee (IACUC), Yonsei Medical Center, Seoul, Korea (IACUC number 2013-0295-1). The animal laboratory was set to 22°C and 50% humidity with a 12-hour light-dark cycle for experiments. Two rats were kept in each cage.

### 2.2. Experimental Procedures

**Figure 1:**
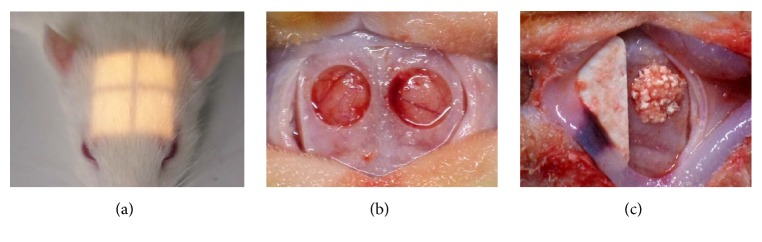
Local irradiation and bone grafting procedure. (a) Field size of 2x2cm (see light window); (b) creating defect (Ø5.0); (c) membrane covered after bone graft.

Animals were classified into four groups according to irradiation and HBO (n = 7): positive control, negative control, HBO-1, and HBO-3 (Table 1). All experimental procedures were conducted under general anesthesia through intraperitoneal injection of an anesthetic cocktail of zolazepam ® (Zoletil, 50 mg/ml, 0.6 ml/kg; Virbac Lab. Carros, France) and xylaxine ® (Rompun, 23.32 mg/ml, 0.4 ml/kg; Bayer Korea, Seoul, South Korea). Rats received localized radiation with a single dose of 12.0 Gy [16, 17] to the calvarial area at 300 kV/12.5 mA using X-RAD 320 (Precision X-Ray; North Branford, CT, USA) and field size 2 × 2 cm. At 4 weeks after irradiation, the calvarial site was shaved before infiltration anesthesia. After exposing the calvarial site, critical size defects were made bilaterally on a sagittal suture (outer diameter 5.0 mm) [17]. A critical size defect is the smallest defect that does not completely heal spontaneously in animals. In general, the critical size defect for the rat calvarial is 8 mm [18]. However, experiments show that complete healing does not occur even at smaller defects, and 5-mm [19, 20] and 4.6-mm [21] defects are also reported as critical size defects. The right side received synthetic bone-graft material (OSTEON™ II Collagen; Genoss, Suwon, South Korea), covered with a membrane (HA collagen membrane; Genoss, Suwon, South Korea) [17, 22, 23]. On the left side, the membrane was applied without a bone graft. Periosteum and skin were sutured using 4-0 silk. Metacam ® (1 mg/kg, once a day for 5 days; meloxicam, Boehringer Ingelheim, Rhein, Germany) and baytril (10 mg/kg/day, once a day for 5 days; Enrofloxacin ®, Bayer, Germany) were administered to prevent infection and pain. The HBO-1 and HBO-3 groups received HBO (2.4 ATA/day, for 6 day/week) for 1 week and 3 weeks, respectively. HBO was gradually applied for 15 minutes under pressure and decompression and maintained at 100% oxygen for 60 minutes at 2.4 ATA. All animals were sacrificed after 6 weeks of bone grafting and surgical sites were removed (Figure 1). Extracted specimens were fixed in 10% formalin for 7 days to prevent damage to the periosteum and dura matter.

### 2.3. Microcomputed Tomography Analysis

**Figure 2:**
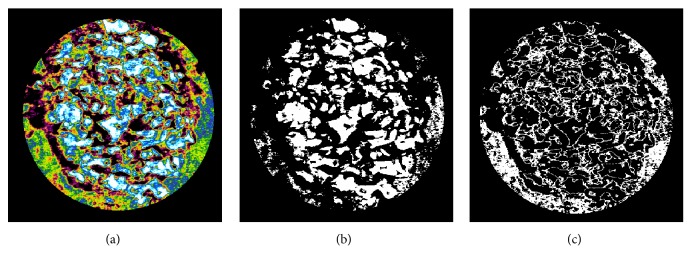
Gray scale of micro-CT analysis in surgical site. (a) Total bone (90-255); (b) residual material (135-255); (c) new bone (90-135).

After fixation 7 days, high-resolution microcomputed tomography (micro-CT, Skyscan1173, Skyscan, Konitch, Belgium) was performed. Specimens were photographed at 100 kV, 100 *μ*A, and pixel size 8.17 *μ*m and analyzed by CTAn software (Skyscan, Aartselaar, Belgium). Analysis was performed by separating residual materials (RM; gray scale 135-255) and new bone (NB; gray scale 90-135) (Figure 2) [17]. Volume of interest was set to 5 mm in diameter and 0.58mm in height. Bone volume (BV; mm3), bone surface (BS; mm2) and bone surface density (BS/TV; mm2/mm3) were analyzed for RM and NB.

### 2.4. Histologic and Histomorphometric Analysis

Fixed specimens were embedded in paraffin after decalcification for 14 days in 5% HCl. Paraffin blocks were serially sectioned at 5-*μ*m thickness through the center of the defect in the coronal plane of the sample. Slides were stained with hematoxylin and eosin followed by light microscopy (BX50, Olympus Co., Tokyo, Japan).

Tissue specimens were processed at magnification 100 and digitized, and total augmented area (TAA; mm^2^), residual material area (RMA; mm^2^), and connective tissue area (CTA; mm^2^) were measured using Adobe Photoshop (Adobe Photoshop CS4, Mountain View, CA, USA). Measured parameters were quantified using Scope EyE 3.6 (TOMORO, Samkyung Co., Seoul, Korea). Blood vessel number counting was performed within the surgical site. Each parameter was expressed as the percent residual material area (%RMA; RMA/TAA*∗*100) and percent new bone area (%NBA; NBA/TAA*∗*100), percent connective tissue area (%CTA; CTA/TAA*∗*100), and percent blood vessel number (%NBV; BNV/TAA*∗*100).

### 2.5. Statistical Analysis

Statistical analysis used IBM SPSS 23.0 (IBM Corp., Armonk, NY, USA) for radiographic and histomorphometric data. Statistical analyses were between positive and negative control groups, with statistical tests between negative controls and experimental groups. Statistical analysis used the Kruskal-Wallis test followed by posttesting using the Mann-Whitney test. The positive control and negative control groups were significantly different at* P* values less than 0.05 and the negative control, and HBO-1 and HBO-3 groups were significantly different at* P* values less than 0.017. Three experiments were performed to determine significance levels (significance level = 5%/number of tests; 0.017 = 5%/3).

## 3. Results

### 3.1. Clinical Observations

A total of 9 animals died due to side effects of general anesthesia after bone grafting. The distribution was 3 rats in the positive control group, 3 in the negative control group, and 3 in the HBO groups. No animals died during the postoperative healing or HBO phases, and the number of dead animals was made identical for further experiments.

### 3.2. Microcomputed Tomography

#### 3.2.1. Positive Control Group vs. Negative Control Group

No significant differences in BV, BS, and BS/TV were detected in graft material on the bone-grafted side. For the positive control group, the BV of new bone was significantly higher than for the negative control group (Table 2). No significant differences were seen in BV, BS, and BS/TV of the new bone from the nongrafted side (Table 3).

#### 3.2.2. Negative Control Group vs. Experimental Group

**Figure 3:**
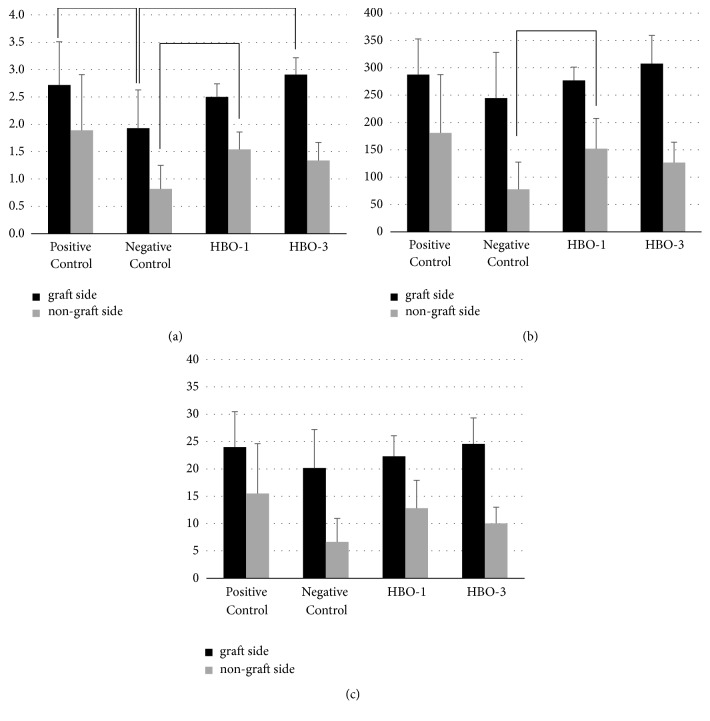
Grafted side and nongrafted side analysis in micro-CT. (a) BV, bone volume in new bone (mm^3^); (b) BS, bone surface in new bone (mm^2^); (c) BS/TV, bone surface density in new bone (mm^2^/mm^3^).

No significant differences were seen in BV, BS, and BS/TV in residual material on the bone-graft side. However the BV of new bone on the grafted side was significantly lower in the negative control group than the HBO-3 group, and there was no significant difference between HBO-1 and HBO-3 group (Table 2, Figure 3). Also, BS and BS/TV did not show any significant difference between HBO-1 and HBO-3 (Table 2, Figure 3). On the nonbone-grafted side, BV and BS of new bone were significantly lower in the negative control than in the HBO-3 groups (Table 3). There was no significant difference between the HBO-1 and HBO-3 groups in BV, BS, and BS/TV on the nongraft side.

#### 3.2.3. Bone-Grafted Side vs. Nongrafted Side

In the positive control group, the BS of the new bone was significantly higher in the bone-grafted side than in the nongrafted side (Figure 3(b)). The BV, BS, and BS/TV values of the bone-grafted side were significantly higher in other groups (Figure 3).

### 3.3. Histological Analysis

**Figure 4:**
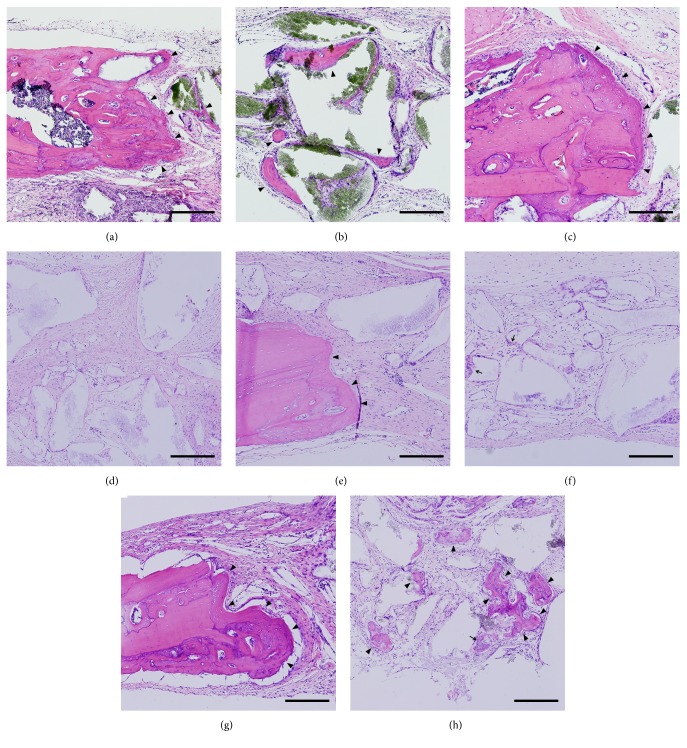
Histological analysis (H-E staining, x200). Scale bar = 200*μ*m. (a, b) Positive control group; (c, d) negative control group; (e, f) HBO-1 group; (g, h) HBO-3 group. Arrowheads, mature bone; arrow, immature woven bone.

In the defect margin region of grafted side, the growth pattern and distance of newly formed bone were higher in the positive control group than in the negative control group (Figures 4(a) and 4(c)). At the center of the defect region, noticeable new bone formation was observed in the positive control group and HBO-3 group (Figures 4(b) and 4(h)). New bone formation in groups increased in the order: negative control, HBO-1, and HBO-3, positive control (Figures 4(c)–4(h)). In the positive control group, almost no new bone was seen, but in the HBO-1 group, osteoclasts were observed with new bone. Island-shaped immature woven bone was clearly observed in the HBO-3 group (Figure 4(h)).

### 3.4. Histomorphometric Analysis

#### 3.4.1. Positive Control Group vs. Negative Control Group

On bone-graft sides, %NBA was significantly increased in the positive control group and %RMA was significantly decreased (Table 4). On the nonbone-graft site, the positive control group had a significantly higher %NBA than the negative control group, while %CTA was significantly lower (Table 5).

#### 3.4.2. Negative Control Group vs. Experimental Group

On the bone-grafted side, %NBA and %BVN were significantly lower in the negative control group than in the HBO-1 and HBO-3 groups (Table 4). On the nongrafted side, %NBA and %BVN were significantly higher in the HBO-1 group than the negative control group (Table 5).

#### 3.4.3. Bone-Grafted Side vs. Nongrafted Side

**Figure 5:**
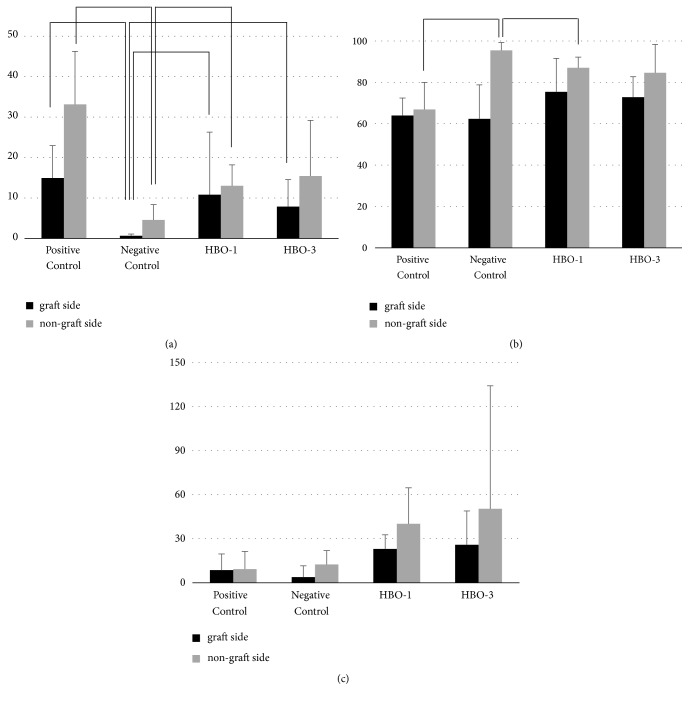
Grafted side and nongrafted side analysis in histomorphometry. (a) %NBA, percent new bone area (mm^2^); (b) %CTA, percent connective tissue area (mm^2^); (c) %BVN, percent bleed vessel number (mm^2^).

The positive control group had a significantly higher %NBA on the nongrafted side than on the bone-grafted side (Figure 5(a)). In the negative control group, %NBA and %CTA were significantly higher in the nongrafted side (Figures 5(a) and 5(b)). In the HBO-1 and HBO-3 groups, %NBA values were higher on the nongrafted side, but the difference was not significant (Figure 5(a)). For the HBO-3 group, %CTA was significantly higher on the nongrafted side and %BVN showed no significant difference among groups but was higher on nongrafted sides (Figures 5(b) and 5(c)).

## 4. Discussion

Radiotherapy and surgical resection are performed for the treatment of head and neck tumors and bone regeneration ability of treatment sites is reduced by radiation therapy [2]. After surgical resection, bone grafting is performed to induce bone regeneration. However, bone grafting alone may have limitations in tissues in which the bone regeneration ability is reduced due to irradiation. This study investigated the effects of HBO on HBO duration and the presence or absence of bone graft.

Comparing the positive control and negative control groups, micro-CT and histomorphometric analysis in grafted side showed that new bone formation was significantly reduced by irradiation. By histomorphometry analysis, remaining grafts were fewer in the positive control group. Micro-CT analysis showed no significant difference in residual materials, but bone volume was less and bone surface was greater in the positive control group than the negative control group. This result indicated that bone metabolism was more active in the positive control group. In the defect margin region, the positive control group actively formed immaturely woven bone, but the negative control group did not. The results of this study confirmed existing results that radiation irradiation significantly degrades bone regeneration ability [2] and we consider that the development of animal models for radiation has been successful.

Comparing the negative control group and experimental groups, the amount of bone formation increased with application of HBO. Micro-CT analysis showed significant differences between the negative control and HBO-3 for BV for new bone, with small differences between the HBO-1 and HBO-3 groups. By histomorphometry analysis, residual material was mostly found in the negative control group, with least in the HBO-1 group. No significant difference was observed between the HBO-1 and HBO-3 groups for any data. These results suggested that HBO was effective in reducing bone loss due to radiation therapy. HBO duration of one week was sufficient.

When bone-grafted and nongrafted sides were compared, the amount of bone formation was significantly higher on the nongrafted than on the grafted side in histomorphometric. BCP is an osteoconductive material that only fills defect volume and does not induce substantial new bone formation. Previous reports showed no significant difference in the amount of new bone when using synthetic bone grafts [24, 25]. However, in this study, significant differences were found in most analyses. HA-coated collagen membranes were used in this study, but the use of a membrane without graft material on the nongraft side seemed to show effects of guided tissue regeneration. Since guided tissue regeneration induces bone-defect healing, bone was actively made in defects without graft material. In addition, osteoconductive material that does not induce osteoinduction in defects with decreased healing potential after radiation appeared to interfere with bone regeneration. The addition of a procedure to apply a graft to defects may lower natural healing potential. However, the success of oral and maxillofacial implants indicates that the presence of sufficient bone at the implant site is the most important factor. Therefore, using osteoconductive material alone to maintain bone volume would be meaningful, even if the rate of new bone formation is slow. In this study, micro-CT showed that bone regeneration on bone-grafted sides was significantly more active in all groups. Conversely, histomorphometry showed no significant differences, but all groups had more bone regeneration on nongrafted sides. However, considering histomorphometry showed no significant differences, it may be regarded as a limited two-dimensional evaluation. Considering these limitations, evaluation by three-dimensional bone analysis is indispensable.

This study evaluated the effect of radiation on bone regeneration in rat calvarial defects of critical size according to HBO duration. In clinical situations, graft material is used to maintain bone-defect volume. Therefore, BCP was applied to defects. Grafted sides showed less new bone formation than nongrafted side. New bone formation increased in groups treated with HBO. In situations where clinical healing potential is reduced, HBO seemed to overcome the disadvantageous bone performance of bone-graft materials. In this study, the sacrifice time of the hyperbaric oxygen group was the same.

The initial effect of HBO was not evaluated, which is a limitation of this study. In a further study, analyzing bone metabolism markers to assess bone remodeling according to HBO timing might confirm HBO effects on bone remodeling.

HBO was evaluated to be effective for bone regeneration in bone tissue with reduced regenerative capacity due to irradiation. In addition, new bone formation with HBO increased in proportion to HBO duration, but was considered to be effective for bone regeneration only with HBO application for one week.

## Figures and Tables

**Table 1:** Study design.

Group		Radiation	Bone Graft	Hyperbaric Oxygen
Positive control	Graft side	-	O	-
	Nongraft side	-	-	-

Negative control	Graft side	O	O	-
	Nongraft side	O	-	-

HBO-1	Graft side	O	O	1 week
	Nongraft side	O	-	1 week

HBO-3	Graft side	O	O	3 weeks
	Nongraft side	O	-	3 weeks

**Table 2:** Volumetric analysis of grafted side in micro-CT (mean±SD). BV, bone volume (mm^3^); BS, bone surface (mm^2^); BS/TV, bone surface density (bone surface/tissue volume; mm^2^/mm^3^); *∗* significant differences compared the positive control and negative control group; † significant differences compared the negative control and HBO-1 group; ‡ significant differences compared the negative control and HBO-3 group.

		Positive control	Negative control	HBO-1	HBO-3
Residual Material	BV	3.13 ± 1.36	3.34 ± 1.62	3.16 ± 0.51	3.57 ± 0.95
	BS	149.59 ± 54.64	121.67 ± 42.71	126.52 ± 10.89	148.21 ± 29.04
	BS/TV	12.53 ± 5.02	10.04 ± 3.64	10.19 ± 1.82	11.84 ± 2.70

New Bone	BV	2.72 ± 0.79 *∗*	1.93 ± 0.70 *∗* ‡	2.5 ± 0.24	2.91 ± 0.31 ‡
	BS	287.43 ± 65.50	244.47 ± 83.75	277.05 ± 24.01	307.72 ± 51.55
	BS/TV	23.99 ± 6.47	20.15 ± 7.04	22.29 ± 3.77	24.56 ± 4.77

**Table 3:** Volumetric analysis of nongrafted side in micro-CT (mean±SD). BV, bone volume (mm^3^); BS, bone surface (mm^2^); BS/TV, bone surface density (bone surface/tissue volume; mm^2^/mm^3^); *∗* significant differences compared the positive control and negative control group; † significant differences compared the negative control and HBO-1 group; ‡ significant differences compared the negative control and HBO-3 group.

		Positive control	Negative control	HBO-1	HBO-3
New Bone	BV	1.89 ± 1.02	0.82 ± 0.43 †	1.54 ± 0.32 †	1.34 ± 0.33
	BS	180.96 ± 106.41	77.73 ± 49.88 †	152.09 ± 55.40 †	126.49 ± 37.41
	BS/TV	15.50 ± 9.12	6.66 ± 4.27	12.80 ± 5.11	10.02 ± 2.97

**Table 4:** Histomorphometric analysis of grafted side (mean±SD). %NBA, percent new bone area (new bone area/total area; mm^2^); %RMA, percent residual material area (residual material area/total area; mm^2^); %CTA, percent connective tissue area (connective tissue area/total area; mm^2^); %BVN, percent blood vessel number (blood vessel number/total area; 1/mm^2^); *∗* significant differences compared the positive control and negative control group; † significant differences compared the negative control and HBO-1 group; ‡ significant differences compared the negative control and HBO-3 group.

	Positive control	Negative control	HBO-1	HBO-3
%NBA	14.94 ± 7.99 *∗*	0.68 ± 0.42 *∗* † ‡	10.83 ± 15.43 †	7.89 ± 6.66 ‡
%RMA	21.12 ± 11.25 *∗*	37.02 ± 16.23 *∗*	13.84 ± 5.27	19.31 ± 12.58
%CTA	63.94 ± 8.44	62.31 ± 16.45	75.33 ± 16.24	72.80 ± 9.91
BVN ratio	8.64 ± 10.99	3.76 ± 7.73 † ‡	23.06 ± 9.60 †	25.87 ± 23.06 ‡

**Table 5:** Histomorphometric analysis of nongrafted side (mean±SD). %NBA, percent new bone area (new bone area/total area; mm^2^); %CTA, percent connective tissue area (connective tissue area/total area; mm^2^); %BVN, percent blood vessel number (blood vessel number/total area; 1/mm^2^); *∗* significant differences compared the positive control and negative control group; † significant differences compared the negative control and HBO-1 group; ‡ significant differences compared the negative control and HBO-3 group.

	Positive control	Negative control	HBO-1	HBO-3
%NBA	33.12 ± 13.12 *∗*	4.58 ± 3.80 *∗* †	13.01 ± 5.15 †	15.44 ± 13.75
%CTA	66.88 ± 13.12 *∗*	95.42 ± 3.80 *∗* †	86.99 ± 5.15 †	84.56 ± 13.75
BVN ratio	9.23 ± 12.05	12.43 ± 9.43 †	40.14 ± 24.63 †	50.45 ± 83.78

## Data Availability

The data used to support the findings of this study are available from the corresponding author upon request.

## References

[1] Vissink A., Jansma J., Spijkervet F. K., Burlage F. R., Coppes R. P. (2003). Oral sequelae of head and neck radiotherapy. *Critical Reviews in Oral Biology & Medicine*.

[2] Doh R., Kim S., Keum K. C. (2016). Postoperative irradiation after implant placement: a pilot study for prosthetic reconstruction. *The Journal of Advanced Prosthodontics*.

[3] Ihde S., Kopp S., Gundlach K., Konstantinović V. S. (2009). Effects of radiation therapy on craniofacial and dental implants: a review of the literature. *Oral Surgery, Oral Medicine, Oral Pathology, Oral Radiology, and Endodontology*.

[4] Schoen P. J., Raghoebar G. M., Van Oort R. P. (2001). Treatment outcome of bone-anchored craniofacial prostheses after tumor surgery. *Cancer*.

[5] Mendenhall W. M. (2004). Mandibular Osteoradionecrosis. *Journal of Clinical Oncology*.

[6] Mainous E. G., Boyne P. J., Hart G. B. (1973). Hyperbaric oxygen treatment of mandibular osteomyelitis: report of three cases. *Journal of the American Dental Association*.

[7] Hallman M., Cederlund A., Lindskog S., Lundgren S., Sennerby L. (2001). A clinical histologic study of bovine hydroxyapatite in combination with autogenous bone and fibrin glue for maxillary sinus floor augmentation: results after 6 to 8 months of healing. *Clinical Oral Implants Research*.

[8] Schlegel K. A., Schultze-Mosgau S., Wiltfang J., Neukam F. W., Rupprecht S., Thorwarth M. (2006). Changes of mineralization of free autogenous bone grafts used for sinus floor elevation. *Clinical Oral Implants Research*.

[9] Hart G. B., Mainous E. G. (1976). The treatment of radiation necrosis with hyperbaric oxygen (OHP). *Cancer*.

[10] Marx R. E. (1983). A new concept in the treatment of osteoradionecrosis. *Journal of Oral and Maxillofacial Surgery*.

[11] Andreassen T. T., Cacciafesta V. (2004). Intermittent parathyroid hormone treatment enhances guided bone regeneration in rat calvarial bone defects. *J Craniofac Surg*.

[12] Edwards M. L. (2010). Hyperbaric oxygen therapy. Part 1: history and principles. *Journal of Veterinary Emergency and Critical Care*.

[13] Drager J., Harvey E. J., Barralet J. (2015). Hypoxia signalling manipulation for bone regeneration. *Expert Reviews in Molecular Medicine*.

[14] Marx R. E., Ehler W. J., Tayapongsak P., Pierce L. W. (1990). Relationship of oxygen dose to angiogenesis induction in irradiated tissue. *The American Journal of Surgery*.

[15] Pedersen T., Xing Z., Finne-Wistrand A., Hellem S., Mustafa K. (2013). Hyperbaric oxygen stimulates vascularization and bone formation in rat calvarial defects. *International Journal of Oral and Maxillofacial Surgery*.

[16] Nussenbaum B., Rutherford R. B., Krebsbach P. H. (2005). Bone regeneration in cranial defects previously treated with radiation. *The Laryngoscope*.

[17] Park K., Hu K., Choi H. (2019). Synergistic effect of hyperbaric oxygen therapy with PTH [1–34] on calvarial bone graft in irradiated rat. *Oral Diseases*.

[18] Takagi K., Urist M. R. (1982). The reaction of the dura to bone morphogenetic protein (BMP) in repair of skull defects. *Annals of Surgery*.

[19] Bosch C., Melsen B., Vargervik K. (1998). Importance of the critical-size bone defect in testing bone-regenerating materials. *The Journal of Craniofacial Surgery*.

[20] Mardas N., Kostopoulos L., Karring T. (2002). Bone and suture regeneration in calvarial defects by e-PTFE-membranes and demineralized bone matrix and the impact on calvarial growth: An experimental study in the rat. *The Journal of Craniofacial Surgery*.

[21] Al-Qutub M., Al-Omar N., Ramalingam S. (2016). Guided bone regeneration using biphasic calcium phosphate with adjunct recombinant human bone morphogenetic protein-2 with and without collagen membrane in standardized calvarial defects in rats: a histologic and biomechanical analysis. *The International Journal of Periodontics & Restorative Dentistry*.

[22] Chang H., Oh S., Oh S., Hu K., Kim S. (2016). Four-week histologic evaluation of grafted calvarial defects with adjunctive hyperbaric oxygen therapy in rats. *Journal of Periodontal & Implant Science*.

[23] Choi H., Park N., Jamiyandorj O. (2012). Improvement of osteogenic potential of biphasic calcium phosphate bone substitute coated with synthetic cell binding peptide sequences. *Journal of Periodontal & Implant Science*.

[24] Escobar T., Almeida e Sousa J., Portela A., Vasconcelos M., Faria de Almeida R. (2014). The effect of a biphasic calcium phosphate on bone healing: a pilot study in rats. *The International Journal of Oral & Maxillofacial Implants*.

[25] Hwang J., Park J., Lee J. (2012). Comparative evaluation of three calcium phosphate synthetic block bone graft materials for bone regeneration in rabbit calvaria. *Journal of Biomedical Materials Research Part B: Applied Biomaterials*.

